# Targeting ATM-deficient CLL through interference with DNA repair pathways

**DOI:** 10.3389/fgene.2015.00207

**Published:** 2015-06-10

**Authors:** Gero Knittel, Paul Liedgens, Hans C. Reinhardt

**Affiliations:** ^1^Department of Internal Medicine, University Hospital of CologneCologne, Germany; ^2^Cologne Excellence Cluster on Cellular Stress Response in Aging-Associated Diseases, University of CologneCologne, Germany

**Keywords:** chronic lymphocytic leukemia, DNA damage response, PARP inhibitor, DNA-PKcs inhibitor, precision medicine

## Abstract

Chronic lymphocytic leukemia (CLL) is the most common form of leukemia in the Western world and accounts for approximately 30% of adult leukemias and 25% of non-Hodgkin lymphomas. The median age at diagnosis is 72 years. During recent years numerous genetic aberrations have been identified that are associated with an aggressive course of the disease and resistance against genotoxic chemotherapies. The DNA damage-responsive proapoptotic ATM-CHK2-p53 signaling pathway is frequently mutationally inactivated in CLL either through large deletions on chromosome *11q* (*ATM*) or *17p* (*TP53*), or through protein-damaging mutations. Here, we focus on the role of ATM signaling for the immediate DNA damage response, DNA repair and leukemogenesis. We further discuss novel therapeutic concepts for the targeted treatment of ATM-defective CLLs. We specifically highlight the potential use of PARP1 and DNA-PKcs inhibitors for the treatment of *ATM*-mutant CLL clones. Lastly, we briefly discuss the current state of genetically engineered mouse models of the disease and emphasize the use of these preclinical tools as a common platform for the development and validation of novel therapeutic agents.

## Background

Genome maintenance is a major challenge for all life on earth. In mammals, genomic integrity is preserved through mechanisms that ensure the faithful transmission of fully replicated and undamaged DNA during each cell division (Hoeijmakers, 2001, 2009). For this purpose, eukaryotic organisms evolved a complex DNA surveillance program: Prior to mitosis, cells progress through G_1_/S-, intra-S and G_2_/M cell cycle checkpoints (Bartek and Lukas, 2007; Reinhardt and Yaffe, 2013). These checkpoints are activated in response to incomplete DNA replication (e.g., due to stalled replication forks), as well as genotoxic damage induced by internal and external sources, such as UV radiation, reactive oxygen species, ionizing radiation (IR) or DNA-damaging chemotherapeutic agents (Weinert, 1998; Zhou and Elledge, 2000; Abraham, 2001; Kastan and Bartek, 2004; Lukas et al., 2004; Bartek and Lukas, 2007). Active checkpoints halt cell cycle progression and thus provide the time necessary to resolve genomic damage (Reinhardt and Yaffe, 2013). If the genotoxic insult exceeds repair capacity, additional signaling cascades, leading to programmed cell death, are activated (Reinhardt and Yaffe, 2013). Thus, DNA damage checkpoints serve as an effective mechanism to provide and maintain genomic stability (Zhou and Elledge, 2000; Kastan and Bartek, 2004; Reinhardt and Yaffe, 2013).

Coherent with a prominent role of the DNA damage response (DDR) in genome maintenance, many DDR-associated genes have been found to be altered in the germline of patients suffering from cancer-prone inherited syndromes, such as *Li-Fraumeni* (*TP53*), *Ataxia telangiectasia* (*ATM*), Seckel syndrome (*ATR*), *Nijmegen breakage syndrome* (*NBS1*), *A-T-like disease* (*MRE11*), *Xeroderma pigmentosum* (XP complementation groups) or familial breast and ovarian cancer (*BRCA1*, *BRCA2*, *RAD51C*) (Frebourg and Friend, 1992; Lavin and Shiloh, 1997; Lehmann, 2003; O'Driscoll et al., 2003; Shiloh, 2003; Taylor et al., 2004; Nevanlinna and Bartek, 2006; Fackenthal and Olopade, 2007; Meindl et al., 2010). Disabling mutations within DDR genes have been proposed to result in a so-called “*mutator phenotype*,” which is thought to drive the runaway proliferation of incipient cancer cells through the accumulation of additional cancer-driving or resistance-causing genomic aberrations (Loeb et al., 2003, 2008; Jiricny, 2006; Jackson and Bartek, 2009; Lord and Ashworth, 2012). While defects in DDR genes appear to facilitate malignant transformation, exploiting these genome-destabilizing alterations for targeted anti-cancer therapy offers a promising therapeutic avenue. In this review, we will focus on cancer-associated defects in ATM-mediated DNA double-strand (DSB) repair and their potential targeting. We will further pinpoint the lack of suitable genetically engineered mouse models of CLL as a critical bottleneck for the rapid preclinical evaluation of novel targeted therapies.

## DNA double strand break repair

**Figure 1:**
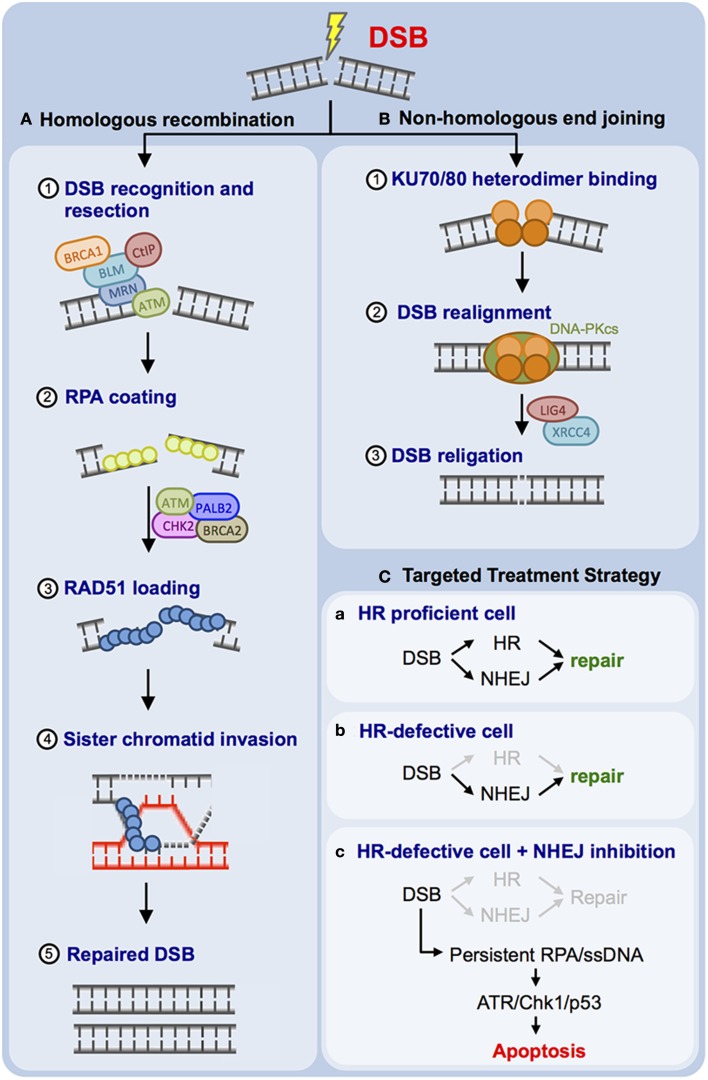
**Mammalian cells employ two principal DNA double-strand break (DSB) repair pathways. (A)** Schematic representation of the error-free homologous recombination (HR) pathway. DSB resection (1), RPA coating (2), RAD51 coating (3), strand invasion (4), and DSB repair are illustrated (5). **(B)** Schematic representation of non-homologous end joining (NHEJ). Ku70/Ku80 binding (1), DNA-PK holo-enzyme assembly and recruitment of additional NHEJ factors, such as LIG4 and XRCC4 (2), as well as DSB religation (3) are illustrated. **(C)** Proposed targeting of HR-defective human cancer through DNA-PKcs inhibition is outlined (for details please refer to the main text).

DSBs can be inflicted by different agents, such as IR and topoisomerase II inhibitors (e.g., etoposide) (Reinhardt and Yaffe, 2009). Mammalian cells use two major DSB repair mechanisms (Figures 1A,B). The error-prone non-homologous end joining (NHEJ) pathway, which does not depend on an intact DNA replication product as a template for repair, can be employed throughout all cell cycle phases (Figure 1B) (Dietlein and Reinhardt, 2014; Dietlein et al., 2014a). NHEJ is primarily used throughout G_1_-phase, when no intact sister chromatid is available as a template for repair. NHEJ-mediated DSB repair relies on the catalytic activity of the protein kinase DNA-PKcs, which is recruited to the break site through physical interactions with the non-catalytic subunits Ku70 and Ku80 (Lees-Miller and Meek, 2003). DNA-PKcs activity mediates the assembly of additional NHEJ factors, such as XRCC4- and Lig4, which facilitate re-ligation of the DSB ends during NHEJ (Lees-Miller and Meek, 2003). Homologous recombination (HR)-mediated DSB repair is the second DSB repair pathway employed by mammalian cells (Figure 1A). HR is an error-free DSB repair mechanism that requires the presence of an intact DNA replication product, which is used as a template. This template dependence leads to a restriction of HR use to late S- and G_2_-phase (Chapman et al., 2012; Dietlein and Reinhardt, 2014; Dietlein et al., 2014a). One of the earliest steps of the HR process is resection of the DSB to create a single-stranded 3′-DNA overhang, which is engaged and coated by the single-stranded DNA (ssDNA)-binding protein RPA (Cimprich and Cortez, 2008; Lyndaker and Alani, 2009). RPA is subsequently replaced by RAD51 in an ATM/CHK2/BRCA1/BRCA2/PALB2-dependent process (Sung and Klein, 2006; San Filippo et al., 2008; Heyer et al., 2010; Krejci et al., 2012). This ssDNA overhang then serves to invade the intact sister chromatid as an intact copy for DNA repair (Sung and Klein, 2006; San Filippo et al., 2008; Krejci et al., 2012). During the HR process, RAD51 fulfills a key role by mediating homology search, strand exchange, and Holliday junction formation (Chapman et al., 2012).

## ATM signaling and the DNA damage response

The proximal DDR kinase ATM, which is mutated in the human cancer-prone disorder *Ataxia telangiectasia* (A-T), is a key regulator of the cellular DDR and essentially controls three different functional outcomes of DDR signaling: cell cycle checkpoints, DNA repair and apoptosis (Reinhardt and Yaffe, 2009; Shiloh and Ziv, 2013). Immediately following the occurrence of a DSB, the trimeric MRN complex, consisting of *M*RE11, *R*AD50 and *N*BS1, is recruited to the site of the lesion (Chapman and Jackson, 2008; Reinhardt and Yaffe, 2013). In parallel, ATM is activated and tethered to the site of the DSB via a physical interaction with the C-terminus of NBS1 (Falck et al., 2005). ATM subsequently phosphorylates histone H2AX on Ser-139. The resulting phospho-H2AX is commonly referred to as γ-H2AX (Rogakou et al., 1998, 1999; Bartek and Lukas, 2007; Reinhardt and Yaffe, 2009). The phosphorylated Ser-139 residue in the C-terminal region of γ-H2AX subsequently binds with high affinity to the phosphopeptide-recognizing BRCT domains of the mediator protein MDC1 (Lee et al., 2005; Stucki et al., 2005; Lou et al., 2006), which in turn is phosphorylated by ATM at multiple residues (Matsuoka et al., 2007). In addition, MDC1 is phosphorylated by the constitutively active Ser/Thr kinase CK2 (Spycher et al., 2008). The resulting phospho-motif is recognized through the phosphopeptide-binding FHA and/or BRCT domains of NBS1 (Chapman and Jackson, 2008; Spycher et al., 2008; Lloyd et al., 2009). This CK2-dependent NBS1 recruitment retains the MRN complex and NBS1-bound ATM at the DSB site (Melander et al., 2008; Spycher et al., 2008). Thus, MDC1, through ATM- and CK2-directed phosphorylation, tethers both the MRN complex and active ATM at the break site, essentially forming an ATM auto-amplification loop.

Coherent with its role in checkpoint signaling and genome maintenance, *ATM* is frequently mutated in various human cancer entities, ranging from solid tumors to lymphomas and leukemias (Haidar et al., 2000; Ripolles et al., 2006; Ding et al., 2008; Waddell et al., 2015). Moreover, bi-allelic loss of *ATM* was shown to be associated with resistance against genotoxic chemotherapy and reduced patient survival (Ripolles et al., 2006; Austen et al., 2007; Skowronska et al., 2012). Recent *in vitro* experiments suggest that ATM is required for the execution of chemotherapy-induced p53-mediated apoptosis (Jiang et al., 2009). Together these data might rationalize why disabling *ATM* alterations are a selected genomic aberration in human neoplastic disease.

Intriguingly, ATM is not only a critical mediator of DNA damage-induced apoptosis, but has also been shown to play a major role in DNA repair, specifically HR-mediated DSB repair, with a less well-characterized role in NHEJ (Luo et al., 1996; Dar et al., 1997; Chen et al., 1999; Morrison et al., 2000; Yuan et al., 2003; Kuhne et al., 2004; Riballo et al., 2004; Xie et al., 2004; Bredemeyer et al., 2006; Shrivastav et al., 2009). Experiments performed with *ATM*-deficient DT40 cells, as well as *A-T* cells derived from patients have shown that these cells display a mild, but distinct HR defect as the result of impaired assembly and functioning of RAD51-associated protein complexes (Morrison et al., 2000; Shiloh, 2003; Yuan et al., 2003). Specifically, a decreased and delayed formation of RAD51 foci was observed in *A-T* cells following IR (Shiloh, 2003; Morrison et al., 2000; Yuan et al., 2003). As detailed above, RAD51 recruitment requires an RPA-coated 3′-single-stranded overhang and thus prior DSB resection. This DSB resection process was shown to be ATM-dependent (Adams et al., 2006; Jazayeri et al., 2006; Myers and Cortez, 2006). Further investigation revealed that ATM is specifically involved in HR-mediated DSB repair during the G_2_-phase of the cell cycle. For instance, it was recently shown that IR-induced sister chromatid exchanges in G_2_ are ATM-dependent (Beucher et al., 2009; Conrad et al., 2011; Jeggo et al., 2011). Furthermore, CtBP-interacting protein (CtIP), which promotes efficient DSB resection during the HR process, recently emerged as an ATM substrate (Shibata et al., 2011). The rather mild DNA repair defect that is observed in *ATM*-deficient cells might be explained by the recent observation that ATM appears to control HR-mediated DSB repair specifically in heterochromatin (HC) regions of the genome (Goodarzi et al., 2008, 2010; Jeggo et al., 2011). These experiments revealed that approximately 85% of IR-induced DSBs are rapidly repaired through a largely ATM-independent process. Approximately 15% of IR-induced DSBs are repaired via a slow-acting repair process that depends on ATM (Goodarzi et al., 2010). Intriguingly, DSBs that undergo delayed repair are mainly restricted to areas of the genome that consist of HC (Goodarzi et al., 2010). It was further shown that ATM directly phosphorylates the HC-building factor KAP-1. This KAP-1 phosphorylation allows HR-mediated DSB repair within HC regions. Furthermore, KAP-1-depletion was demonstrated to rescue the DSB repair defect induced by *ATM* deficiency (Goodarzi et al., 2008, 2010; Jeggo et al., 2011). Altogether these data strongly suggest that the apoptosis-evading effect of *ATM*-deficiency, which likely stems from insufficient p53 activation, is associated with a potentially druggable HR defect.

## Defective ATM-dependent DSB repair as a potential therapeutic target in CLL

Chronic lymphocytic leukemia (CLL) is a lymphoproliferative disorder that accounts for approximately 30% of adult leukemias and 25% of non-Hodgkin lymphomas (NHL) (Hallek and Pflug, 2010). It is the most common form of leukemia in the western world with an incidence rate of 4-5/100.000 (Hallek and Pflug, 2010). CLL is a disease of the elderly with <10% of the patients being <40 years of age and a median age at diagnosis of 72 years (Hallek and Pflug, 2010). CLL is extraordinarily heterogeneous in its clinical manifestation, treatment response and course. Some patients live for decades and do not require any therapeutic intervention, while others suffer from rapidly progressive and refractory disease (Cramer and Hallek, 2011). It is this extraordinary heterogeneity, which makes treatment of CLL especially challenging. To date, no curative therapy exists besides allogeneic stem cell transplantation, for which most patients do not qualify due to age or reduced performance status. However, it is important to note that we are witnessing a paradigm shift in the treatment of CLL with new, targeted agents recently approved (e.g., ibrutinib, idelalisib), or being evaluated in advanced approval trials (ABT-199). These novel agents interfere directly with B cell receptor signaling (ibrutinib—BTK Inhibitor, idelalisib—PI3Kδ Inhibitor), or relieve repression of the pro-apoptotic proteins BAX and BAK through BCL2 blockade (ABT-199) (for an excellent review, please refer to Thompson et al., 2015).

A hallmark feature of CLL cells is an extraordinarily high frequency of genomic aberrations, which can be documented in more than 80% of CLL patients (Dohner et al., 2000; Di Bernardo et al., 2008; Crowther-Swanepoel et al., 2010; Ouillette et al., 2010). Moreover, the failure of all conventional chemotherapies to induce long-lasting remissions strongly suggests that the apoptosis-mediating DDR is crippled in CLL. The genomic instability of CLL cells is reflected by a number of cytogenetic abnormalities that occur recurrently in CLL. For instance, deletions of the short arm of chromosome 17 (*del(17p)*) are found in 5–8% of chemotherapy-naïve patients. These deletions almost always include band *17p13*, where the prominent tumor suppressor gene *TP53* is located. CLL patients carrying a *del(17p)* clone show marked resistance against genotoxic chemotherapies that cannot be overcome by the addition of anti-CD20 antibodies in the context of state of the art chemo-immunotherapy (Hallek et al., 2010). Among cases with confirmed *del(17p)*, the majority show mutations in the remaining *TP53* allele (>80%) (Seiffert et al., 2012). Disabling *TP53* mutations are enriched in chemotherapy-treated patients, suggesting that an inactivation of the pro-apoptotic ATM-CHK2-p53 signaling cascade is selected for in CLL (Puente et al., 2011; Quesada et al., 2011).

Deletions of the long arm of chromosome 11 (*del(11q)*) can be found in approximately 25% of chemotherapy-naïve patients with advanced disease stages and 10% of patients with early stage disease (Zenz et al., 2010; Puente et al., 2011; Quesada et al., 2011). These deletions frequently encompass band *11q23* harboring the *ATM* gene. A subset of approximately 40% of patients carrying a *del(11q)* clone display inactivating mutations of the second *ATM* allele and these cases show a poor chemotherapy response, reminiscent of what has been described for *TP53*-defective CLLs (Austen et al., 2007). In addition, patients carrying a *del(11q)* clone typically show rapid progression, and reduced overall survival (Seiffert et al., 2012). As for *TP53*, disabling *ATM* mutations are enriched in chemotherapy-treated patients, again suggesting that an inactivation of the pro-apoptotic DDR is selected for in CLL (Puente et al., 2011; Quesada et al., 2011). It remains to be seen whether the novel agents, including ibrutinib, idelalisib, ABT-199, obinotuzumab or lenalidomide might overcome the reduced prognosis of *del(17p)*/*TP53* and *del(11q)*/*ATM* altered cases.

Recently, two novel potential therapeutic approaches to specifically treat ATM-deficient neoplastic disease have emerged from *in vitro* and *in vivo* experiments performed in different laboratories.

As ATM is involved in HR-mediated DSB repair (Figure 1A), it was proposed that repression of NHEJ, the second prominent DSB repair pathway employed by mammalian cells, might display selective toxicity against ATM-defective cells while sparing healthy cells (Figure 1C) (Gurley and Kemp, 2001; Jiang et al., 2009; Reinhardt et al., 2009; Riabinska et al., 2013; Dietlein and Reinhardt, 2014; Dietlein et al., 2014a,b). Early experiments performed with *ATM*^−/−^ and *PRKDC*^−/−^ (encoding DNA-PKcs) mice revealed that double knockout animals undergo early embryonic lethality (E7.5), while single knockout animals were born alive (Xu et al., 1996; Gao et al., 1998; Gurley and Kemp, 2001). These data revealed a robust synthetic lethal interaction between *ATM* and *PRKDC* and suggest that pharmacological interception of DNA-PKcs signaling might be detrimental to *ATM*-defective *del(11q)* CLLs. Consistent with this hypothesis, combined depletion of *Atm* and *Prkdc* in *Myc*-driven transplanted murine lymphomas led to a massive sensitization of these lymphomas against the anthracycline doxorubicine (Figure 1C) (Jiang et al., 2009; Reinhardt et al., 2009). Pharmacological DNA-PKcs inhibition has recently been evaluated in preclinical systems (Figure 1C). DNA-PKcs repression with the ATP-competitive small molecule inhibitor KU-0060648 resulted in robust induction of apoptosis of *ATM*-defective cells *in vitro* (Riabinska et al., 2013). Furthermore, KU-0060648 displayed substantial cytotoxicity against *Atm*-depleted *Myc*-driven murine lymphomas, while *Atm*-proficient lymphomas were entirely resistant (Riabinska et al., 2013). The authors next extended their observations to freshly isolated CLL cells. While KU-0060648 displayed marked single agent activity against *del(11q)* CLL cells, cytogenetically normal cells did not show any apoptosis following drug exposure (Riabinska et al., 2013). Further analyses revealed that DNA-PKcs inhibition in *ATM*-defective cells prevents effective DSB repair (Riabinska et al., 2013). On a molecular level, the authors showed that KU-0060648-exposed *ATM*-defective cells initiate DSB resection and accumulate RPA-coated ssDNA intermediates. These structures ultimately trigger apoptotic cell death through activation of the RPA/ATRIP/ATR/CHK1/p53/Puma apoptotic signaling cascade (Riabinska et al., 2013). Further experiments showed that not only *ATM*-deficiency, but also other HR-impairing genetic aberrations, such as *BRCA1*-, *BRCA2*-, *FANCD2*- or *RAD50* mutations were associated with DNA-PKcs dependence (Dietlein et al., 2014b). Together these data suggest that DNA-PKcs inhibitors either as single agents or in combination with DSB-inducing chemotherapeutics might be a viable treatment option for *del(11q)* CLLs. Intriguingly, Celgene has developed CC-115, a small molecule compound that is currently being evaluated in phase I/II clinical trials as a combined DNA-PKcs/mTOR inhibitor for the treatment of both solid tumors and hematological malignancies, including CLL (ClinicalTrials.gov identifier: NCT01353625).

**Figure 2:**
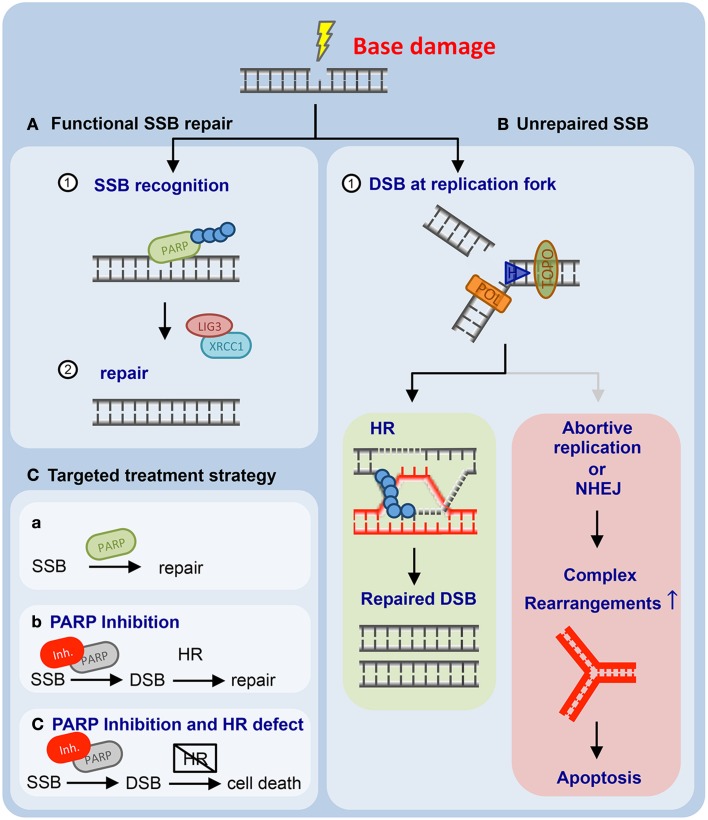
**Mammalian cells employ base excision repair to resolve single-strand breaks (SSBs) and non-helix-distorting base modifications. (A)** Unperturbed base excision repair (BER) requires PARP1 and LIG3 and XRCC1. **(B)** PARP1 inhibition leads to the accumulation of genotoxic lesions that are subsequently repaired through homologous recombination (HR)-mediated DNA repair (left panel). If HR-mediated DNA repair is unavailable, PARP1 inhibitor-induced genotoxic damage accumulates and ultimately results in apoptotic cells death (right panel). **(C)** Proposed targeting of HR-defective human cancer through PARP1 inhibition is outlined (for details please refer to the main text).

A second potential therapeutic approach for *ATM*-defective human neoplastic disease has recently emerged from preclinical model systems. Different groups have shown that PARP1 inhibitors display selective toxicity against *ATM*-defective cells (Williamson et al., 2012; Gilardini Montani et al., 2013; Kubota et al., 2014) (Figures 2A–C). PARP1 inhibitors have recently gained the attention of the biomedical community, as they have been demonstrated to selectively eradicate *BRCA1*- or *BRCA2-deficient* cells and tumors (Figure 2C) (Bryant et al., 2005; Farmer et al., 2005). PARP1 inhibitor treatment was shown to induce DNA damage in *BRCA1* or *BRCA*2-proficient and -deficient cells (Farmer et al., 2005). However, only *BRCA1* or *BRCA2*-defective cells were sensitive to PARP1 inhibition, while *BRCA1/2* wildtype cells were PARP1 inhibitor-resistant (Farmer et al., 2005). Subsequent experiments revealed that additional DNA repair-disabling cancer-associated mutations in genes such as *RAD51*, *RAD54*, *DSS1*, *RPA1*, *NBS1*, *ATR*, *ATM*, *CHK1*, *CHK2*, *FANCD2*, *FANCA*, or *FANCC* were also associated with PARP1 inhibitor sensitivity (McCabe et al., 2006). These results motivated additional experiments that tested the hypothesis that *ATM* deficiency could be an actionable genetic alteration that might be susceptible to PARP1 inhibition. In this regard, four pieces of data have recently been published. First, RNA interference-mediated *ATM* repression was shown to sensitize MCF-7 and ZR-75-1 breast cancer cells (ER-positive, HER2-negative, *BRCA1/2* wildtype, *TP53* wildtype) to the PARP1 inhibitor olaparib (Gilardini Montani et al., 2013). Second, a focused gastric cancer cell line screen revealed that low ATM protein expression significantly correlated with olaparib sensitivity (Kubota et al., 2014). A further characterization revealed that pharmacological- or RNA-interference-mediated repression of ATM kinase activity enhanced olaparib sensitivity in gastric cancer cell lines with parallel depletion or inactivation of p53 (Kubota et al., 2014). In addition to these solid tumor entities, PARP inhibitors have also been evaluated in hematological malignancies. In mantle cell lymphoma xenograft transplants it was recently shown that animals carrying lymphomas lacking both *ATM* and *TP53* (UPN2) displayed significant olaparib sensitivity. Similarly, in mice transplanted with lymphomas lacking *ATM* and one copy of *TP53*, olaparib induced a significant survival gain. In contrast, mice transplanted with ATM- and p53-proficient lymphomas (JVM-2), or lymphomas with isolated p53 inactivation (HBL-2), did not derive a survival benefit from olaparib (Williamson et al., 2010, 2012). Lastly, proliferating primary *ATM*-deficient CLL cells were shown to display increased olaparib sensitivity, compared to *ATM*-proficient counterparts (Weston et al., 2010). Both genetic and pharmacological experiments validated that this effect was ATM-dependent (Weston et al., 2010). Furthermore, the authors employed a murine xenograft model of an *ATM*-mutant mantle cell lymphoma cell line to demonstrate a significantly reduced lymphoma burden and an increased survival of animals following olaparib treatment *in vivo* (Weston et al., 2010). Altogether, these data suggest that PARP1 inhibition might be a useful strategy for the treatment of refractory *ATM*-defective CLLs (Figure 2C).

## Perspectives

One of the biggest hurdles in preclinical CLL research and preclinical development of targeted CLL therapeutics is the lack of mouse models that faithfully mimic the genetic events leading to human CLL development. Although several models exist (for an excellent review, please refer to Simonetti et al., 2014), none of these models truly recapitulates the multistep leukemogenesis typically observed in CLL patients. Specifically the high-risk aberrations, such as *Tp53*- or *Atm* deletion/mutation are thus far not sufficiently recapitulated. Although *Tp53*^−/−^ mice have been crossed with *Eμ-Tcl1* transgenic animals, the resulting compound-mutant *Eμ-Tcl1*;*Tp53*^−/−^ mice carried a homozygous germline deletion of *Tp53*, which limits their use as a preclinical model to mirror somatic *del(17p)* or *TP53*-mutation in CLL (Liu et al., 2014). Of note, *Eμ-Tcl1*;*Tp53*^−/−^ mice develop B-CLL substantially earlier than *Eμ-Tcl1* mice with an early appearance of CD5^+^/IgM^+^ B cells in the spleen (Liu et al., 2014). These animals display an aggressive course of disease development, as well as a drug resistance phenotype reminiscent of human *del(17p)* CLL (Liu et al., 2014). These data suggest that a B cell-specific conditional *Tp53* deletion, for instance through the use of *Cd19-Cre*^*ERT*2^ deleter mice on the *Eμ-Tcl1* background, might be a useful experimental strategy to faithfully mimic clonal evolution of p53-defective CLL. In addition, B cell-specific conditional *Atm* deletion using the recently published *Atm*^*fl*^ allele (Zha et al., 2008) should be performed with *Cd19-Cre*^*ERT2*^ deleter mice in the *Eμ-Tcl1* background. Furthermore, it is desirable to translate recent large scale CLL genome sequencing data into preclinical platforms. For instance, generation of mice carrying a B cell-specific *Myd88*^*L265P*^ mutation, which has recently been described as a potential early driver lesion in CLL (Landau et al., 2013), should be pursued (Figure S1).

### Conflict of interest statement

The authors declare that the research was conducted in the absence of any commercial or financial relationships that could be construed as a potential conflict of interest.

## Supplementary material

Figure S1 **Schematic proposal of the use and early integration of genetically engineered mouse models for the development of novel CLL therapeutics**. Recent large-scale chronic lymphocytic leukemia (CLL) genome sequencing efforts have unraveled the identity of numerous potential driver mutations in CLL. With this genomic information in hand, we propose the generation of novel genetically-engineered mouse models of CLL to serve as a preclinical platform for the identification and validation of novel CLL therapeutics.
