# Supplementary figures and images for: Targeting ATM-deficient CLL through interference with DNA repair pathways
Source: Front Genet. 2015 Jun 10;6:207. doi: 10.3389/fgene.2015.00207 (PMC4461826; doi:10.3389/fgene.2015.00207)

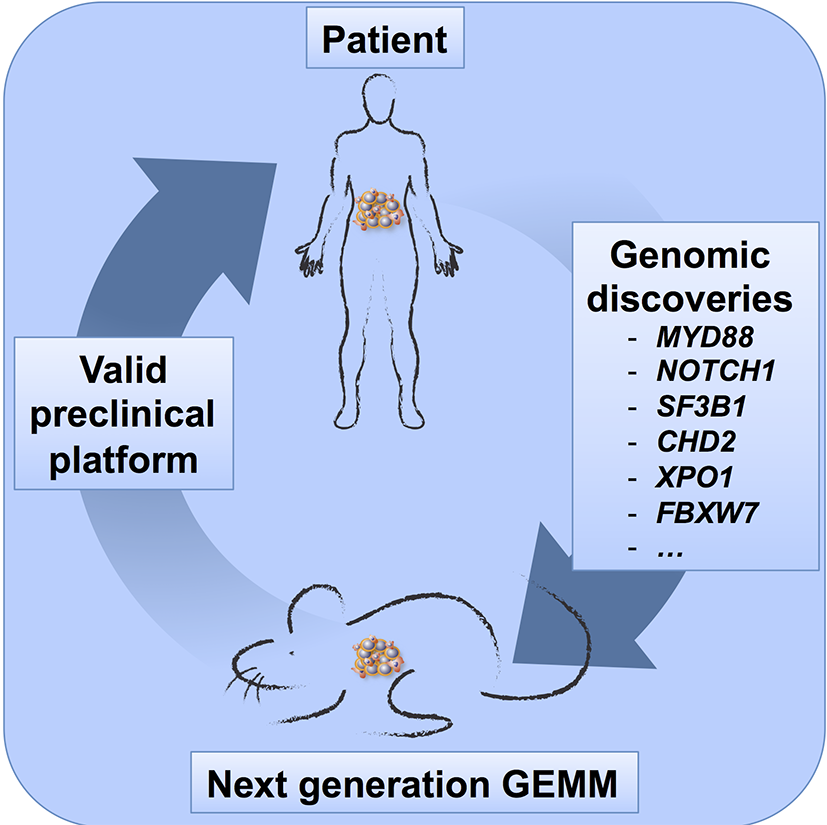

Supplement: Figure S1 — Schematic proposal of the use and early integration of genetically engineered mouse models for the development of novel CLL therapeutics. Recent large-scale chronic lymphocytic leukemia (CLL) genome sequencing efforts have unraveled the identity of numerous potential driver mutations in CLL. With this genomic information in hand, we propose the generation of novel genetically-engineered mouse models of CLL to serve as a preclinical platform for the identification and validation of novel CLL therapeutics. [file Image1.TIF]
